# ‘Thinking outside the box’: advanced geriatric nursing in primary health care in Scandinavia

**DOI:** 10.1186/s12912-019-0350-2

**Published:** 2019-07-02

**Authors:** Erika Boman, Ann-Louise Glasberg, Rika Levy-Malmberg, Lisbeth Fagerström

**Affiliations:** 1 Department of Nursing and Health Sciences, University of South-Eastern Norway, Drammen, Norway; 2 Department of Nursing, Åland University of Applied Sciences, Neptunigatan 17, 22100 Mariehamn, Åland Finland; 3 Department of Nursing, University of Applied Sciences, Novia, Vaasa, Finland; 4 Faculty of Education and Welfare Studies, Åbo Akademi University, Vaasa, Finland

**Keywords:** Advanced practice nursing, Gerontology, Geriatric nurse practitioner, Older persons, Primary health care, Qualitative content analysis

## Abstract

**Background:**

Older people are frequent users of primary health care (PHC) services. PHC services have been critiqued, mainly regarding limited accessibility and continuity of care. In many countries, investment in nurse practitioners (NPs) has been one strategy to improve PHC services. In the North of Europe, the NP role is still in its infancy. The aim of this study was to explore the feasibility of introducing geriatric nurse practitioners (GNPs) in PHC in Scandinavia, from multiprofessional and older persons’ perspectives.

**Methods:**

The study had a qualitative design, including 25 semi-structured interviews with nurses, nurse leaders, physicians, politicians and older persons from several communities in Scandinavia. The material was analysed by means of qualitative content analysis.

**Results:**

The results highlight current challenges in health services for the older population, i.e. comorbid older patients with complex care needs aging in place, lack of competent staff, and organisational challenges. The results present an envisioned GNP scope of practice in health services for the older population, including bringing advanced competence closer to the patient, an autonomous role including task-shifting, and a linking role. The results also present factors influencing implementation of the GNP role, i.e. GNP competence level, unclear role and scope of practice, and openness to reorganisation.

**Conclusions:**

The results indicate that it is feasible to implement the GNP role in primary health care in Scandinavia. Notwithstanding, there are factors influencing implementation of the GNP role that should be considered.

**Electronic supplementary material:**

The online version of this article (10.1186/s12912-019-0350-2) contains supplementary material, which is available to authorized users.

## Background

The postmodern era is characterized by longer life expectancy. Nevertheless, growing old does not necessarily mean living more years in good health [1]. Older people are frequent users of primary health care (PHC) services [2, 3]. A decrease in patient satisfaction among older persons has been noted, and criticism has been voiced, mainly regarding limited accessibility and continuity of care in PHC [2, 4]. Thus, it is suggested that new, innovative models of PHC delivery for older people are developed.

In many countries, a shift of tasks from physicians to nurse practitioners (NPs) has been one strategy to alleviate shortages of staff in PHC. In countries with a long history of the NP role, such as Canada, USA, Australia, Holland and the United Kingdom, NPs are authorized to work at a high level of advanced practice, including advanced health assessments, ordering tests and medical treatment for minor illnesses. In some countries, NPs also have authority to issue prescriptions [5, 6]. Studies indicate that nurse-led care has a positive effect on patient satisfaction, and has led to reduced hospital and/or nursing home admissions and mortality [7–9]. In addition, NPs in ambulatory care could lower mean health services costs per consultation [10]. However, as the results from previous research are not conclusive, larger and more rigorous research in the field is required [c.f. [9, 10].

In the North of Europe, the NP role was introduced more recently. Sweden was the first country to offer a NP master degree program in 2003, followed by Finland in 2005 [11]. However, task shifting to NPs is still limited. In the North of Europe, Finland is the only Nordic country where NPs, with limited prescription rights, are authorised to work at high levels of advanced practice in PHC [6]. Nevertheless, studies indicate that NPs in PHC can constitute a resource to increase access to care and enhance continuity [7, 8, 12]. Development of the role is though restricted by limited independence and lack of an explicit definition of the NP role, as well as demarcation in relation to responsibility and roles among colleagues [8]. Anyhow, top-level managers and politicians in Scandinavia have anticipated that nursing competence on an advanced level will be needed to meet older persons’ complex needs [13, 14]. Nevertheless, research on advanced practice nursing in the care of older people from a Nordic perspective is scarce. The aim of this study was to explore the feasibility of introducing geriatric nurse practitioners (GNSs) in primary health care (PHC) in Scandinavia, from multiprofessional and older persons’ perspectives. The research questions were:

1. What are the overall current challenges in health services for the aged?
2. What is the visionary GNP scope of practice in health services for the aged?
3. What factors may influence implementation of the GNP role?

## Methods

### Context

This qualitative interview study was performed in Scandinavia, i.e. Sweden, Norway and Denmark, including the Faroe Islands. The population in these countries is getting older and according to the population prognosis for the North of Europe, the share of the population above the age of 80 will reach 8.6% in 2040, as compared to the 2013 level of 4.8% [15]. In Scandinavia, the health care system is mainly taxation based, and all citizens have equal access to services. There are split responsible authorities in health service provision. In general, specialist/hospital care is provided by counties (Denmark and Sweden) or regional health authorities (Norway), and the municipalities are responsible for primary and social care [16].

### Participants

The study has it origin in a project comprising a joint master’s degree program in advanced geriatric nursing, equivalent to the NP designation. Five students from Scandinavia (two from Sweden, one from Norway, one from Denmark and one from the Faroe Islands) interviewed one older person, one registered nurse, one nurse leader, one physician and one politician in their home country (i.e. 25 interviews in total) (Table 1). The sampling technique was purposeful, a common sampling technique in qualitative research to capture individuals who are especially knowledgeable about or experienced with the phenomenon of interest [17]. The healthcare professionals were recruited in cooperation with the managers in the region where the GNP students were currently working. The older persons were recruited through local senior citizen associations and politicians through local municipal assemblies or similar. The inclusion criterion was that the participants were to have an interest in inquiries concerning care of older persons.

**Table 1:** Description of participants

Participants (n)	Age min–max (mean)	Years in profession min–max (mean)
Older persons (5)	70–85 (76.0)	
Nurses (5)	47–65 (53.8)	15–43 (26.5)
Nurse leaders (5)	43–63 (56.0)	4–18 (11.4)
Physicians (5)	47–67 (53.8)	4–46 (23.0)
Politicians (5)	51–72 (62.3)	12–50 (29.4)

### Data collection

The interviews were semi-structured. An interview guide was developed in cooperation with the students’ supervisors (i.e. two of the authors of this article: A-L.G. and L.F.). Interview questions are found in Additional file 1. Before the interviews, the participants received oral and written information, including the International Council of Nurses’ definition of NPs [5]. Written informed consent was collected from the participants. The interviews were performed in the native language of the participants and lasted between 25 and 60 min. The interviews were recorded and then transcribed verbatim in the original language.

### Analysis

For analysing, manifest qualitative content analysis inspired by Elo and Kyngäs [18] was performed. The analysis was performed in NVivo12 (QRS International). The transcribed interviews were imported into the software and read several times. In the next step meaning units were identified, i.e. units of the text that were “large enough to be considered as a whole and small enough to be kept in mind as a context for meaning unit during the analysis process” (Elo and Kyngäs [18], p.109). In the next step the material was coded, i.e. each meaning unit was assigned a heading, to later be organised in domains related to the three research questions. Then, by collapsing content that were similar or dissimilar, sub-categories and categories were created (exemplified in Table 2).

**Table 2:** Analysis; examples of meaning units, sub-categories and categories

The current challenges in health services for older population		
Meaning units	Sub-category	Category
The physicians cannot handle it all, we have to raise our level to handle the current needs of the aging population.	Medical competence	Lack of competent staff
It happens that we don’t have an on-call physician. Then we have to contact the hospital … If we would have a physician here the patients would get treatment earlier, and would not have to travel to the hospital (that is hours away)	Medical competence	Lack of competent staff
Firstly, we have no geriatrician, a doctor, and that it is very odd… how serious is the task taken...? I think of doctors and hospital, who is engaging in these old people, geriatric patients…	Competence in gerontology and geriatrics	Lack of competent staff
When it comes to care for the older population there is a need… a need of education in geriatrics… everyone working should have it… it goes without saying… it is the foundation.	Competence in gerontology and geriatrics	Lack of competent staff
This… collaboration between these different levels of care and different care givers… There we have a big problem.	Collaboration	Organisational challenges
The challenge is also that you lack a really good cooperation with the physicians. With some physicians there is good cooperation, with others there is not, and then it is like running the forehead against a wall.	Collaboration	Organisational challenges

The first author (EB) made a first, preliminary version of the results. The results were then discussed and validated at several meetings, to finally be confirmed in the research group.

### Ethics

In Norway, approval to perform the study was obtained from the Norwegian Centre for Research Data (Norsk senter for forskningsdata, NSD; Ref. nr. 37,218/2). Ethical approval was not needed in the other countries, i.e. approval was necessary only if the research involved patients. For permission to interview staff, approval was collected from each of the organizations included in the study.

## Results

Data analysis resulted in nine categories and twenty-one sub-categories organised in three domains corresponding to the research questions (Table 3).

**Table 3:** Summary of results

Domain	The current challenges in health services for older population		
Categories	Care needs of comorbid older patients aging in place	Lack of competent staff	Organisational challenges
Sub-categories	Complex care needs	Medical competence	Collaboration
	Loneliness and lack of social support	Competence in gerontology and geriatrics	Continuity
			Resources
Domain	The envisioned GNP scope of practice in health services for the older population		
Categories	Advanced competence closer to the patient	Autonomous role including task-shifting	Linking role
Sub-categories	Competence to meet the needs of geriatric patient	Autonomous role with focus on assessments	Link between patient, nurse and physician
	Combining the nursing and medicine paradigm	Task-shifting	Link between patient and care
	Being positioned close to the patients		Link to evidence-based practice
Domain	Factors influencing the implementation of the GNP role		
Categories	GNP competence level	Unclear role and scope of practice	Openness to reorganisation
Sub-categories	Doubts about GNP competence level	Rights and obligations	Changing traditional systems
	Taking on increased responsibility and gaining trust	GNP models	Allocation of resources

### The current challenges in health services for the older population

The domain included three categories: Care needs of comorbid older patients aging in place, Lack of competent staff and Organisational challenges.

### Care needs of the comorbid older patients aging in place

The category includes caring for older patients living with complex care needs in their homes or communities. It was concluded from the interviews that there has been a shift from ‘aging in nursing homes’ to ‘aging in place’. One nurse leader said: ‘So it’s only the sickest sick coming in (to nursing homes) now’. Many older persons aging in place have complex care needs and are in need of regular contact with health care services. However, it was said that it could be challenging for the older person to find one’s way around in the health care system, not knowing who to contact with different kinds of health problems. Another concern was that health care today is more about illness and deficiencies than about focusing on the strengths and abilities of the older person. A nurse said: ‘There has been a lot of focus on illness and on the lack of resources, rather than focusing on what the individual actually still can master and manage in their own home or in their daily life’.

In addition, aging in place with complex care needs is related to loneliness and lack of social support. One nurse leader said: ‘It is loneliness that shatters many older people today… loneliness and lack of support in their everyday life, it is not just illness…’.

### Lack of competent staff

Lack of medical competence was expressed as a current challenge in PHC. There is a lack of physicians and/or long distances to medical services, meaning that the older patients have to wait and/or travel to have an assessment and treatment. Lack of available medical support in PHC was also said to result in unneeded hospital admissions.

Lack of competence in gerontology and geriatrics was also expressed by many of the respondents. Competence in gerontology and geriatrics was said to be a prerequisite to get an understanding of the older person and to offer high quality and holistic care. Challenges in recruiting competent staff were mentioned.

### Organisational challenges

The health care system in the Scandinavian countries is split between responsible authorities. Lack of collaboration between the parties was mentioned by several of the respondents. A politician stated that they are concerned about patients that are in need of different services, risking to find themselves outside everyone’s responsibility. Lack of collaboration between different service providers were also found in expressions like: ‘Just getting coordinated with the medication… what medication they took at home (care provided by the municipality) and what they had in the hospital (care provided by the county)… there are so many mistakes, almost every time, and that is a big problem’, one physician said. In addition, a politician said: ‘We had worst imaginable outcome… a patient in need of regular home services was at home without any service for two whole days after a hospital stay before someone remembered…’. Lack of collaboration between responsible authorities could also mean that the older persons were referred to a number of different health care institutions instead of getting coordinated care.

Lack of collaboration between health care professionals was also mentioned, especially between nurses and physicians. There was also criticism pointed towards the health care organisation for being rigid in respect of supporting traditional roles and hierarchical structures, and sometimes hindering doing what was understood to be in the best interest for the patient. One politician said: ‘We need the services to be more flexible, not as rigid as they have been, so that we quickly can adjust our services, instead of saying that this is the framework, this is the box, and everyone should fit into the box. We must be able to think outside the box and take on the challenges.’

Another organisational challenge was lack of continuity. One physician stated: ‘Sometimes we over-treat’, and referred to the fact that they do not get to know the older patients, who all have different problems but not all problems are related to diseases. ‘Sometimes we try to treat the normal aging process’, the physician continued. Another example of lack of continuity was lack of follow-up. ‘Lack of follow-up is very much the case. Treatment, medication or something else is initiated, but there is no follow-up’, one physician said. Regular reviews of the older patients’ pharmaceutical treatment were requested.

Lack of resources was mentioned as another challenge. To a great extent, practice is regulated by a set timetable. ‘The clock controls many things, and there is little opportunity to find some extra time for the patient, for example if one sees something else appearing than expected’, one nurse said. A physician said: ‘Everything today is seen from an economic cost perspective. --- Everything should be done efficiently and that leads to a short circuit; if we are only focusing on an illness and getting the patient out, then he comes back again.’ A politician stated that more resources should be invested in technical solutions supporting communications to specialist care, making it possible to avoid sending the patient back and forth.

### The envisioned GNP role and scope of practice in health services for the older population

The domain included three categories: Advanced competence closer to the patient, Autonomous role including task-shifting and Linking role.

### Advanced competence closer to the patient

GNPs were assumed to have extended competence, beyond registered nurses, and more competence in gerontology and geriatrics than junior doctors have. A physician said: ‘Junior doctors are getting a lot of responsibility out in the districts, and are supposed to make assessments of complex situations, but the young physicians are often more into what is seen as fancy and extravagant, and do not have the experience to assess a complex situation in geriatric care.’

GNPs were understood to be able to combine the nursing and medicine paradigm. They are to develop competence within the field of medicine, but are still nurses. Several of the respondents stated that many can find it easier to talk to a nurse compared to a physician. ‘In a way, I think you can speak more freely with a nurse’, an older person said. In addition, it an advantage that nurses, in general, are more familiar with the patients and their history.

The respondents expressed the hope that GNPs would be positioned close to the patients. ‘I think you have to work close to the patients, close to the users to be able to see what the needs are’, a physician said. A politician expressed concerns about GNPs being caught behind a desk instead of working close to the patients: an unwanted scenario.

### Autonomous role including task-shifting

GNPs were expected to be able to work autonomously in their scope of practice. One politician said: ‘For me it is evident that they will be able to do it (work autonomously) if the midwifes can. If you can go through pregnancy with only two or three visits to the physician to check your health status, and then give birth without any meddling by a physician… then we can do it in geriatrics as well’. A need for nurse-led clinics to meet the complex care needs of geriatric patients was also mentioned in the interviews.

The GNP role was assumed to focus on assessments. A first assessment based on knowledge in gerontology and geriatrics to get the overall picture was emphasised. One nurse leader said: ‘It is about making a qualified assessment from the beginning, an advanced assessment in the beginning to get a comprehensive picture… it is about medical conditions, but also including social factors and psychological factors’. Based on this comprehensive assessment the older patients’ care planning would be set up.

GNPs were also envisioned to suggest further assessments, for example laboratory tests and x-ray, and to initiate some treatments. The GNP role was suggested to complement physicians, but also to include task-shifting, i.e. taking on tasks that traditionally have been a part of the physician’s role. One physician referred to lack of physicians and said: ‘… as it has been done in Finland, it is possible to move some of the tasks to well-educated nurses’. GNPs were also envisioned to have the role to observe, follow up and evaluate care that is given.

### Linking role

The GNPs were expected to take on a linking role between patient, nurses and physicians. It was claimed that there are health concerns of the patients that fall between the competence of registered nurses and physicians. ‘GNPs can fill the gap between physicians and nurses’ one older person said, and a politician said: ‘Yes, it would be a link between registered nurses and physicians.’

The GNPs were also envisioned to take on a linking role between patient and health services, taking on a case management role especially for patients with comorbidity and complex care needs. ‘For example the comorbid patients with complex care needs… these patients could be assigned to a GNP, being a fixed contact… being at the heart of things, being in contact with other service providers’, a nurse leader said.

GNPs were also seen as a link to evidence-based practice. Having a Masters Degree, the GNPs were expected to support nursing staff in their professional development based on evidence-based practice. It was also said that nursing staff should be able to consult the GNP. ‘They (nursing staff) are in situations, fumbling through trial and error, using unnecessary energy because they can’t get in contact with the physician, who anyhow doesn’t grasp the situation they are in... I absolutely think that it would have been fantastic if we would have the possibility to have one or two NPs in home care’, one nurse said.

### Factors influencing the implementation of the GNP role

The domain included three categories: GNP competence level, Unclear role and scope of practice and Openness to reorganisations.

### GNP competence level

The respondents raised concerns about the redistribution of tasks from physicians to nurses. The concerns, expressed by several of the respondents, regarded whether the GNPs would be ready to meet the extensive demands of assessing and initiating treatment of older patients. It was alleged that if their training was not good enough, there would be risk of under and/or over treatment of older people. One physician stated: ‘I think it is nice that nurses bring different suggestions to the table, they often express wise thoughts. But the one having the right to prescribe, that should be the physician’.

On the other hand, another physician said: ‘It should not be a problem as long as one has made a medical assessment – if one has made assessments, one is also responsible for them.’ Responsibility was said to be related to competence. ‘If your competence is not recognized… You can take on responsibility in some respect, but you also have to earn it. If you don’t get it, you won’t be able to act… that is my experience’, a leader said regarding proving acquired competence and gaining trust from co-workers and from patients, as well as knowing one’s own limitations.

### Unclear role and scope of practice

The GNP’s authorities and responsibilities were found unsettled. The distribution of tasks, foremost between physicians and the GNPs, was understood as unclear. In addition, there were questions about differences and similarities compared to other specialized nurses. It was stated that before implementing GNP positions, the scope of practice must be well defined and informed throughout the care chain.

It was also said that there has to be well-defined work descriptions and clarifications of in which care situations the GNP competence could best be used. This information should be defined and communicated within the unit and throughout the organization. ‘We have to get a work description so we know what that person (GNP) can be used for… get a specification in which situations we can call you’, a nurse said.

### Openness to reorganisations

Implementing a new nursing role in the health care system was said to offer an opportunity to change the traditional organisational structures. ‘It offers new thinking… thinking differently… mix the cards again’, one leader said. However, it was said that it requires courage to stand up against a well-established system. ‘It requires that you dare to stand up against the current model and dare to try something new’, a politician said. Bold leaders supporting the GNPs were understood to be an important driving force. This, especially since task-shifting may threaten hierarchical systems. ‘You can say that this is about a shift in paradigm and it is about power. And I can see that there is a certain… a certain fear of losing power in some professional categories’, a leader said.

The implementation of the GNP role in the health care system was expected to also require an allocation of resources. If GNPs are to take on additional responsibility, including task-shifting, they should be economically compensated. The respondents declared a need to prove the necessity of GNPs to convince politicians and key persons in health care organizations to establish GNP positions. It was mentioned that higher salaries intended for GNPs would not be beyond dispute, especially among nurses with numerous years of experience and possibly competence, albeit informal, comparable to that of the formally educated GNPs.

## Discussion

The aim of this study was to explore the feasibility of implementing GNPs in PHC in Scandinavia. Feasibility says something about the likelihood of being able to do something, the practicality of a proposed plan, and can be understood as an evaluation and analysis of the potential of a proposed intervention [19]. The interviews reveal that there are common challenges in health services assigned for the older population in Scandinavia, challenges that also are present internationally [20, 21]. Looking into the results, it seems feasible that the envisioned GNP scope of practice correspond to current challenges in health services assigned for the older population. Notwithstanding, there are factors influencing the implementation of the GNP role that should be considered.

Doubts regarding the implementation of the GNP role were expressed in the results. Making advanced assessments, diagnosing and initiating treatment, including prescriptive authority could solve some practical problems, but at the same time there were concerns about this part of the GNP role (see also [12, 22]). In addition, the legislative rights for nurses to prescribe medication on an advanced level in Scandinavia are limited [6, 8]*.* To utilize the GNP competence fully, as in many other countries [6], policymakers need to initiate regulatory reforms to sanction independent advanced practice nursing roles.

It was not stated that GNPs should replace physicians, but to complement the team (see also [22]). GNPs need to be able to consult physicians if necessary [7, 12, 23]. If successful, GNPs could take on a linking role, promoting teamwork and cooperation within and between different professions and organizations [22, 23, 25]. The implementation of GNPs could further favour continuity in care as the GNPs would have the prerequisites of meeting the older people in their homes, having a case management role and with the foundation in a caring perspective contributing to creating the groundwork for holism and ‘home as a health promotive context’ (cf. [23, 24, 26, 27]). It should also be mentioned that the establishment of GNP positions offers a clinical career opportunity for nurses, entailing greater authority and responsibility, and development of personal competencies [22], a possibility that may attract nurses and also tackle the lack of competence in the field (cf. [13, 28, 29]).

In any event, to succeed there is a need to develop standardized definitions, a clear conception of the GNP role and scope of practice, and a minimum education level [22, 23, 30]. Here the Nordic NP model [11] that emphasises advanced clinical skills and a synthesis of curing and caring, can make a valuable contribution. The model is understood to align with the results of this study, describing the potential role and responsibilities transferable to meet the problems in care of older persons, and serving as a framework for implementing GNP positions in PHC.

To succeed with the implementation of GNP roles it is further implied that there is a need of openness to reorganisations. In line with previous research [31], the leaders are said to be in a critical position to influence the success of organizational change. The leaders must guide, motivate, support and communicate, and use various leadership styles to bring about actual change. It is not just a question about introducing a new routine, but about establishing a cultural change in patterns, values and attitudes [32, 33].

The results suggest that it would be feasible to introduce GNPs in PHC as a mean to address the current challenges, in line with previous research (e.g. [9, 10, 24]). However, it would be naïve to believe in that the implementation of the GNP role in the health care system would solve every problem. If other interventions are not carried out, it is for example probable that older persons still will report lack of social support, there will still be organisational challenges devoted to split authorities, and even though GNPs could reduce the lack of competent staff, there will still be a need for physicians, preferably geriatricians. Nonetheless, the health care system will need to evolve to respond to patient desires and needs, including social and medical care provided by non-physicians like GNPs. Older adults reporting loneliness and other psychosocial factors, in combination with medical problems, cannot rely solely on medicine.

There are several methodological concerns to be considered in this study. The interviews were performed by students with limited experience in performing qualitative research. Nonetheless, they had long work experience as nurses, including long experience in interviewing patients in their daily work. The students were prepared and supervised in performing research interviews before the data collection begun, and were in in close contact with the supervisors, who have considerable experience in qualitative research, during the data collection. The interview guide was developed in cooperation with the supervisors.

Another limitation was that the interviews were performed in different countries with different languages and cultures, and deficient language skills and cultural understanding may have affected the results. Consequently, the interviews and the results were discussed several times in the student and research groups to strengthen credibility. It is further considered a strength that different professionals were interviewed, and that older persons also were included. However, numbers of representatives from each country was small. Nonetheless, the results suggest that there are similar challenges in health services for the aged in Scandinavia. Further, there seems the be an overall consensus on the envisioned GNP role and scope of practice including factors that may influence the implementation of this advanced practice nursing role. No prominent deviation in responses between countries, nor between groups of respondents could be found. However, all group of respondents were not representing all sub-categories, i.e. the older persons did not mention organisational challenges.

## Conclusion

It seems feasible that the GNP role and scope of practice broadly correspond to current challenges in health services assigned for the older population in the Scandinavian countries. Notwithstanding, there are factors influencing the implementation of the GNP role that should be considered. It is of importance to clarify the NP competence level, the role and authorities and responsibilities, as well as to prove the necessity to stimulate openness to reorganisation before implementation. Furthermore, policymakers have to initiate regulatory reforms to sanction advanced practice nursing. Only then, the full competence of the GNP can be applied and used in its full scope of practice.

## Additional file

Additional file 1: Interview guide. (DOCX 16 kb)

## Data Availability

The datasets generated and/or analysed during the current study are not publicly available due lack of consent of sharing raw material, but parts of the material can be available from the corresponding author on reasonable request.

## Abbreviations

GNP: Geriatric Nurse Practitioner
NP: Nurse Practitioner
NSD: Norwegian Center for Research Data
PHC: Primary Health Care
