# Supplementary material for: ‘Thinking outside the box’: advanced geriatric nursing in primary health care in Scandinavia
Source: BMC Nurs. 2019 Jul 2;18:25. doi: 10.1186/s12912-019-0350-2 (PMC6604267; doi:10.1186/s12912-019-0350-2)
Supplement: Supplementary file 1 — Interview guide. (DOCX 16 kb) [file 12912_2019_350_MOESM1_ESM.docx]

Supplement

**Interview guide**

1. How would you describe the current problems/shortcomings in the care and treatment of older people in primary health care/municipal health services?
2. What are your thoughts on NP role/model that is presented in the fact sheet? What are your thoughts on NPs taking greater responsibility in the care of older people?
3. What are your thoughts on NP role/responsibilities in care of older people?
4. What do you see is needed to implement the new NP model?
5. Is there anything that makes you hesitant/doubtful when it comes to the new NP model?
6. How do you think you can support the development of NPs in your organization/ your municipality?
7. How do you think your colleagues/others in your team respond to NPs?
